# Markers of systemic inflammation are positively associated with influenza vaccine antibody responses with a possible role for ILT2(+)CD57(+) NK-cells

**DOI:** 10.1186/s12979-022-00284-x

**Published:** 2022-05-26

**Authors:** Emilie Picard, Sarah Armstrong, Melissa K. Andrew, Laura Haynes, Mark Loeb, Graham Pawelec, George A. Kuchel, Janet E. McElhaney, Chris P. Verschoor

**Affiliations:** 1 Health Sciences North Research Institute, 41 Ramsey Lake Road, Rm 32033, Sudbury, ON P3E 5J1 Canada; 2 Department of Medicine, Dalhousie University, Halifax, NS Canada; 3 UConn Center On Aging, University of Connecticut School of Medicine, Farmington, CT USA; 4 Department of Pathology and Molecular Medicine, McMaster University, Hamilton, ON Canada; 5 Department of Immunology, University of Tübingen, Tübingen, Germany; 6 Northern Ontario School of Medicine, Sudbury, ON Canada

**Keywords:** Influenza vaccine response, Inflammation, ILT2 + CD57 + NK cells, IL-6, Older adults, Frailty

## Abstract

**Background:**

With increasing age, overall health declines while systemic levels of inflammatory mediators tend to increase. Although the underlying mechanisms are poorly understood, there is a wealth of data suggesting that this so-called “inflammaging” contributes to the risk of adverse outcomes in older adults. We sought to determine whether markers of systemic inflammation were associated with antibody responses to the seasonal influenza vaccine.

**Results:**

Over four seasons, hemagglutination inhibition antibody titres and ex vivo bulk peripheral blood mononuclear cell (PBMC) responses to live influenza viruses assessed via interferon (IFN)-γ/interleukin (IL)-10 production, were measured pre- and 4-weeks post-vaccination in young adults (*n* = 79) and older adults randomized to standard- or high-dose inactivated vaccine (*n* = 612). Circulating tumour necrosis factor (TNF), interleukin (IL)-6 and C-reactive protein (CRP) were also measured pre-vaccination. Post-vaccination antibody titres were significantly associated with systemic inflammatory levels; specifically, IL-6 was positively associated with A/H3N2 titres in young adults (Cohen’s d = 0.36), and in older high-dose, but not standard-dose recipients, all systemic inflammatory mediators were positively associated with A/H1N1, A/H3N2 and B titres (d = 0.10–0.45). We further show that the frequency of ILT2(+)CD57(+) CD56-Dim natural killer (NK)-cells was positively associated with both plasma IL-6 and post-vaccination A/H3N2 titres in a follow-up cohort of older high-dose recipients (*n* = 63). Pathway analysis suggested that ILT2(+)CD57(+) Dim NK-cells mediated 40% of the association between IL-6 and A/H3N2 titres, which may be related to underlying participant frailty.

**Conclusions:**

In summary, our data suggest a complex relationship amongst influenza vaccine responses, systemic inflammation and NK-cell phenotype in older adults, which depends heavily on age, vaccine dose and possibly overall health status. While our results suggest that “inflammaging” may increase vaccine immunogenicity in older adults, it is yet to be determined whether this enhancement contributes to improved protection against influenza disease.

**Supplementary Information:**

The online version contains supplementary material available at 10.1186/s12979-022-00284-x.

## Background

The likelihood of serious illness and injury increases dramatically as we age, especially after 65 years [1]. The risk of serious outcomes of respiratory infection with influenza is particularly concerning because older adults suffer higher rates of hospitalization and mortality [2, 3], in addition to immediate complications such as myocardial infarction [4] and delayed post-influenza functional impairment [5]. This is also true with respect to acute care and outcomes related to SARS-COV-2 infection [6], and for certain symptoms of what is commonly referred to as “long-COVID” [7, 8]. As observed with SARS-COV-2 [9], antibody [10] and cell-mediated [11] responses to the seasonal influenza vaccine wane with age, as does the effectiveness to prevent hospitalization, although this varies depending on subtype, season, and overall health status [12–14]. Of the three major influenza (sub)types included in the seasonal vaccine, A/H3N2 breakthrough tends to result in more hospitalizations [15] and mortality [16] in older adults, partly due to limited vaccine-induced antibody production [17, 18], and especially in years of vaccine mismatch [19].

A number of studies have provided evidence towards understanding the underlying causes of poor vaccine antibody responses and effectiveness in older adults, such as early life exposure (so-called original antigenic sin) [20], repeated vaccination [21, 22], and B-cell defects [23] and diversity within the T-cell repertoire [24]. While this is useful for our understanding of age-related defects in influenza vaccine responses, it does little to help explain the heterogeneity in antibody responses within the older adult population. One particular area that has been understudied is the low-level chronic inflammation which commonly increases with age (ie. “inflammaging” [25]), and plays a prominent role in age-related immune dysfunction of B-cells [26], monocytes [27] and macrophages [28]. Further, we have shown that circulating levels of inflammatory mediators (ie. C-reactive protein [CRP]) are inversely correlated with responses to the varicella-zoster virus vaccine [29], while others have shown them to be related to the risk of adverse outcomes in older adults, such as depression [30], cardiovascular disease [31], frailty [32], and all-cause mortality [31, 33].

In the following study, we sought to estimate the association of circulating inflammatory mediators – tumor necrosis factor (TNF), interleukin (IL)-6 and CRP – with responses to standard or high-dose influenza vaccination in young and older adults, hypothesizing that greater inflammation would correlate with poor responsiveness. In contrary, our findings indicate that the levels of these factors are positively correlated with antibody titres post-vaccination, most consistently in older, high-dose recipients. We further show that this relationship may be at least partially mediated by natural killer (NK)-cells, which are positively associated with both post-vaccination antibody titres and plasma IL-6 levels.

## Results

### Inflammatory mediators are increased with age and are associated with participant BMI and frailty

**Fig. 1:**
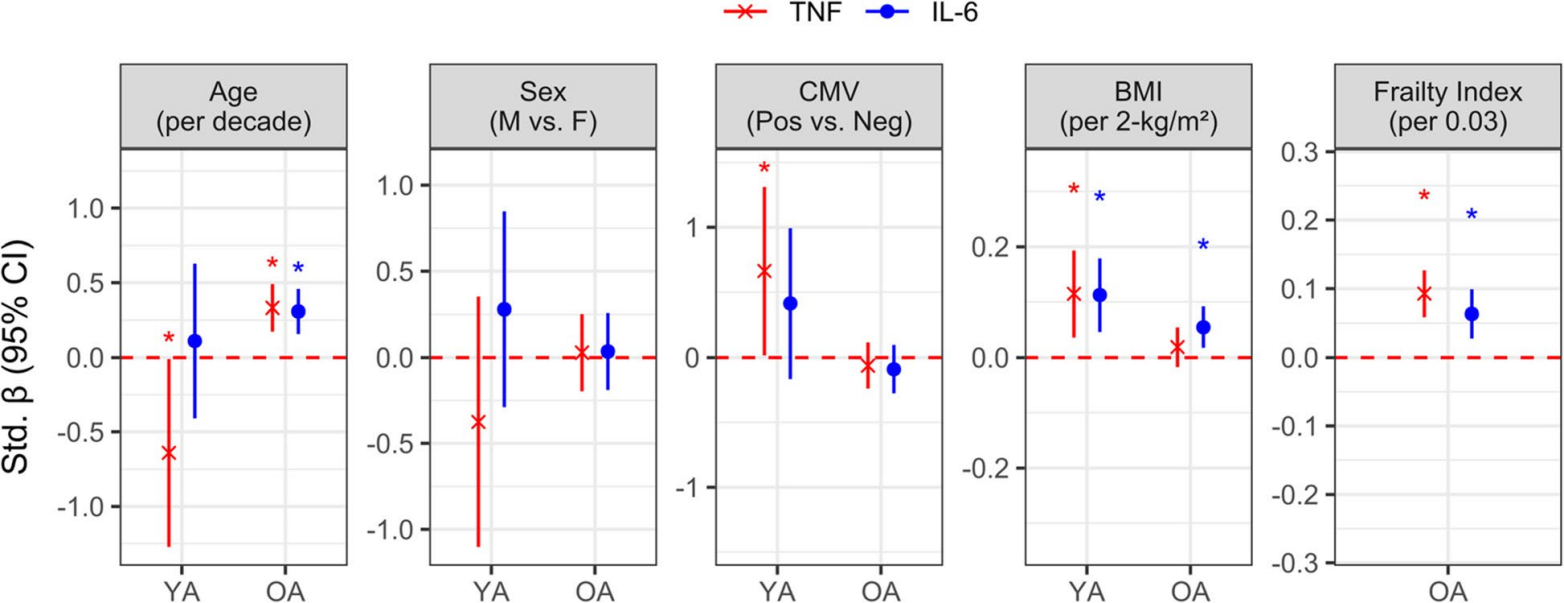
Associations between pre-vaccination plasma TNF and IL-6 levels with participant demographics. In older (OA) and young (YA) adults, natural-log transformed TNF or IL-6 was regressed on age, sex, CMV serostatus, BMI and frailty (OA only), and standardized coefficients (β) and 95% confidence intervals for each independent variable are presented. Coefficients are relative to the change or contrast described in brackets, and significance is indicated by an asterisk and when the 95% confidence interval does not cross the red, dotted line

In the parent vaccination trial, data and biosamples were collected over four influenza seasons from 612 older adults, aged 65 to 96, and 79 young adults, aged 21 to 41; summary statistics on these cohorts can be found in Table 1. Significantly more older adults were cytomegalovirus (CMV)-seropositive as compared to young adults, and plasma levels of both TNF and IL-6 were higher. In older adults, both TNF and IL-6 increased significantly with age and frailty, and IL-6 increased with body mass index (BMI); in young adults, both TNF and IL-6 were significantly higher with BMI and TNF was higher with CMV-seropositivity (Fig. 1). For both age groups, CRP was only associated with BMI: for older participants, the odds ratio for medium vs. low concentration was 1.33 (95% CI: 1.06, 1.67) per 2-kg/m^2^; for young, the odds ratio for medium and high vs. low CRP was 1.34 (95% CI: 1.07, 1.68) and 1.46 (95% CI: 1.13, 1.91) every 2-kg/m^2^, respectively.

**Table 1:** Summary of participants enrolled in each year of the study and pre-vaccination levels of inflammatory mediators

	**Older adults**	**Young adults**	
	**(*N* = 612)**	**(*N* = 79)**	
**Age**	76 (7.4)	30 (4.9)	***
**Sex**			
Female	410 (67.0%)	58 (73.4%)	
Male	202 (33.0%)	21 (26.6%)	
**BMI (kg/m**^**2**^**)**	28 (4.87)	28 (7.25)	
Missing	3 (0.5%)	10 (13%)	
**CMV serostatus**			***
Negative	287 (46.9%)	62 (78.5%)	
Positive	325 (53.1%)	17 (21.5%)	
**Frailty Index**	0.11 (0.073)		
Missing	2 (0.3%)		
**Dose**			***
High-dose	296 (48.4%)	0 (0%)	
Standard-dose	316 (51.6%)	79 (100%)	
**Site**			
HSNRI	356 (58.2%)	40 (50.6%)	
UCHC	256 (41.8%)	39 (49.4%)	
**Year**			
2014/15	106 (17.3%)	19 (24.1%)	
2015/16	175 (28.6%)	20 (25.3%)	
2016/17	174 (28.4%)	20 (25.3%)	
2017/18	157 (25.7%)	20 (25.3%)	
**TNF (pg/ml)**			***
Mean (SD)	12.1 (4.53)	8.53 (1.62)	
Median [Min, Max]	11.1 [2.87, 43.8]	8.45 [5.10, 12.7]	
Missing	3 (0.5%)	1 (1%)	
**IL-6 (pg/ml)**			***
Mean (SD)	8.37 (43.0)	1.60 (1.36)	
Median [Min, Max]	2.70 [0.464, 633]	1.07 [0.193, 7.08]	
Missing	3 (0.5%)	1 (1%)	
**CRP (mg/L)**			
Low (< 5)	429 (70.1%)	54 (68.4%)	
Medium (5–10)	100 (16.3%)	7 (8.9%)	
High (10 +)	41.0 (6.7%)	9 (11.4%)	
Missing	42 (7%)	9 (11%)	

Unless specified otherwise, continuous data is summarized as the mean and standard deviation, while categorical data is the count and frequency. Significance determined by either Wilcoxon rank-sum or Chi-square test, where applicable, *** *p* < 0.001. Note, the frailty index was not measured in young adults

### Associations between pre-vaccination systemic inflammatory mediator levels and vaccine responses differ by age, dose and response type

Both pre- and post-vaccination geometric mean titres against the circulating seasonal strain of A/H1N1, A/H3N2, and B were higher in younger than older adults, although the fold-increase of high-dose older recipients exceeded that of young (Supplementary Table 1); these latter findings have been previously reported [10]. Similar trends were observed for pre- and post-vaccination levels of secreted interferon (IFN)-γ as well as IL-10 following live A/H3N2 stimulation (Supplementary Table 1), which we have also previously reported [11].

**Fig. 2:**
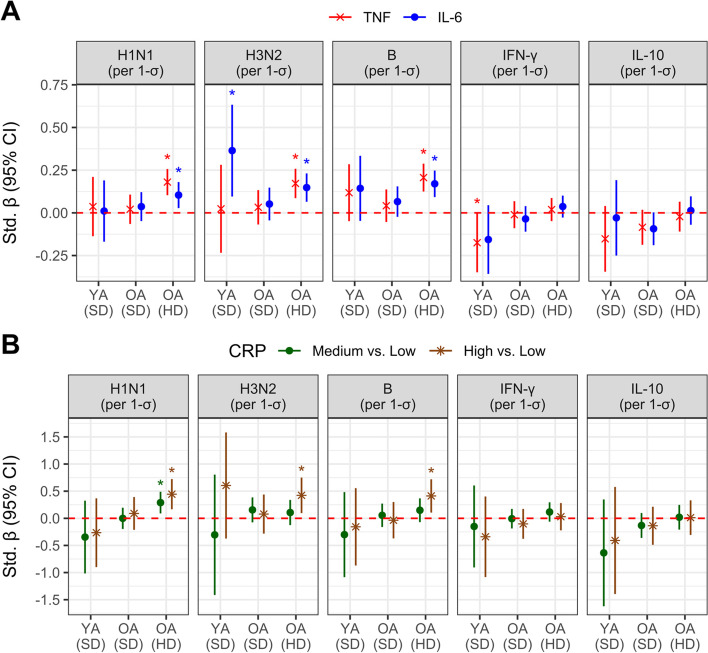
Systemic inflammation and the response to influenza vaccination in young (YA) and older (OA) adults, stratified by vaccine dose (standard [SD] or high [HD] dose). Natural log-transformed antibody titres against the seasonal circulating strain of influenza A/H1N1, A/H3N2 and B, and PBMC IFN-γ and IL-10 secretion in response to ex vivo A/H3N2 challenge were measured at 4-weeks post-vaccination and regressed against pre-vaccination natural-log transformed TNF or IL-6 (**A**), or CRP level categories (**B**), adjusting for pre-vaccination antibody/CMI response and additional covariates; both the outcome and TNF/IL-6 were standardized to facilitate cross-comparison. Standardized coefficients (β) and 95% confidence intervals for TNF, IL-6 and CRP (either the Medium vs. Low, or High vs. Low contrast) are presented, and significance is indicated by an asterisk and when the 95% confidence interval does not cross the red, dotted line

Levels of systemic inflammatory mediators were positively correlated with antibody responses post-vaccination, depending on both age and vaccine dose. In young adults, associations between inflammatory mediators and post-vaccination antibody titres were mostly non-significant; however, a relatively strong and significant positive correlation between IL-6 and A/H3N2 titres was observed (Std. β [95% CI] = 0.36 [0.096, 0.633]; Fig. 2A), as was the difference between participants with high CRP relative to low (0.61 [-0.37, 1.58]; Fig. 2B). For older recipients of the high-dose vaccine, significant positive associations were observed for plasma inflammatory mediator levels with A/H1N1, A/H3N2 or B antibody titres post-vaccination: for TNF and IL-6, a 1-SD increase was associated with 0.10- to 0.21-SD increase in post-vaccination titres (Fig. 2A), and for CRP, the increase for participants with medium (ie. 5–10 mg/L) and high (ie. 10 + mg/L) levels was as much as 0.29- and 0.45-SDs relative to those with low (ie. < 5 mg/L) concentrations, respectively (Fig. 2B). These associations remained significant even after adjusting for frailty (Supplementary Fig. 1), which we have previously shown to be positively correlated with post-vaccination antibody titres [10]. When TNF, IL-6 and CRP were modelled together, the significant positive associations with post-vaccination antibody titres in high-dose recipients remained for TNF, regardless of virus (sub)type, whereas for IL-6 and CRP, effect sizes were reduced such that significant associations were evident only for B and A/H1N1 titres, respectively (Supplementary Table 2); interestingly, frailty lost its association with antibody titres when TNF, IL-6 or CRP were added as a covariate (Supplementary Fig. 1). In contrast to the above, when examining the additive effect of TNF and IL-6 using interaction analysis, a significant increase in post-vaccination titres was evident for standard- and high-dose older recipients that exhibited high levels of both mediators, as compared to low to medium levels, particularly for A/H3N2 (difference in standardized estimated marginal means, high vs. low: standard-dose = 0.370, *p* < 0.001; high-dose = 0.385, *p* < 0.05) (Supplementary Fig. 2). Importantly, this analysis suggested that the association between inflammatory mediators and antibody titres in high-dose recipients is approximately linear, whereas for standard-dose recipients it is non-linear and only evident at the highest levels of inflammation.

As a secondary analysis, we also compared the association of TNF and IL-6 with vaccine responses in older and younger women (Supplementary Fig. 3A) and men (Supplementary Fig. 3B) separately. Although some differences were evident, they were for the most part nominal. Using interaction analysis, the association between IL-6 and A/H1N1 antibody responses was slightly higher in female standard-dose recipients (β_interaction_ [95% CI] = 0.15 [0.29, 0.01]), while the association of IL-6 and B antibody responses was lower in female high-dose recipients (-0.33 [-0.11, -0.55]). In young adults, associations between TNF and A/H3N2 antibody responses were lower in females (-0.54 [-0.01, -1.07]).

Unlike antibody titres, post-vaccination levels of both of secreted IFN-γ and IL-10 following live A/H3N2 stimulation appeared to be associated with lower plasma inflammatory mediator levels, although most estimates were not significant. For standard-dose older recipients, inverse associations were observed for the levels of secreted IL-10 with plasma TNF and IL-6, albeit neither significant (Std. β [95% CI]: TNF = -0.084 [-0.189, 0.018]; IL-6 = -0.093 [-0.189, -0.003]) (Fig. 2A). For young adults, secreted IFN-γ was inversely associated with TNF (-0.175 [-0.348, -0.001]), as was IL-6, but not significantly (-0.156 [-0.357, 0.045]) (Fig. 2A).

### NK-cell phenotype correlates with both systemic inflammation and vaccine antibody responses in older adults

**Fig. 3:**
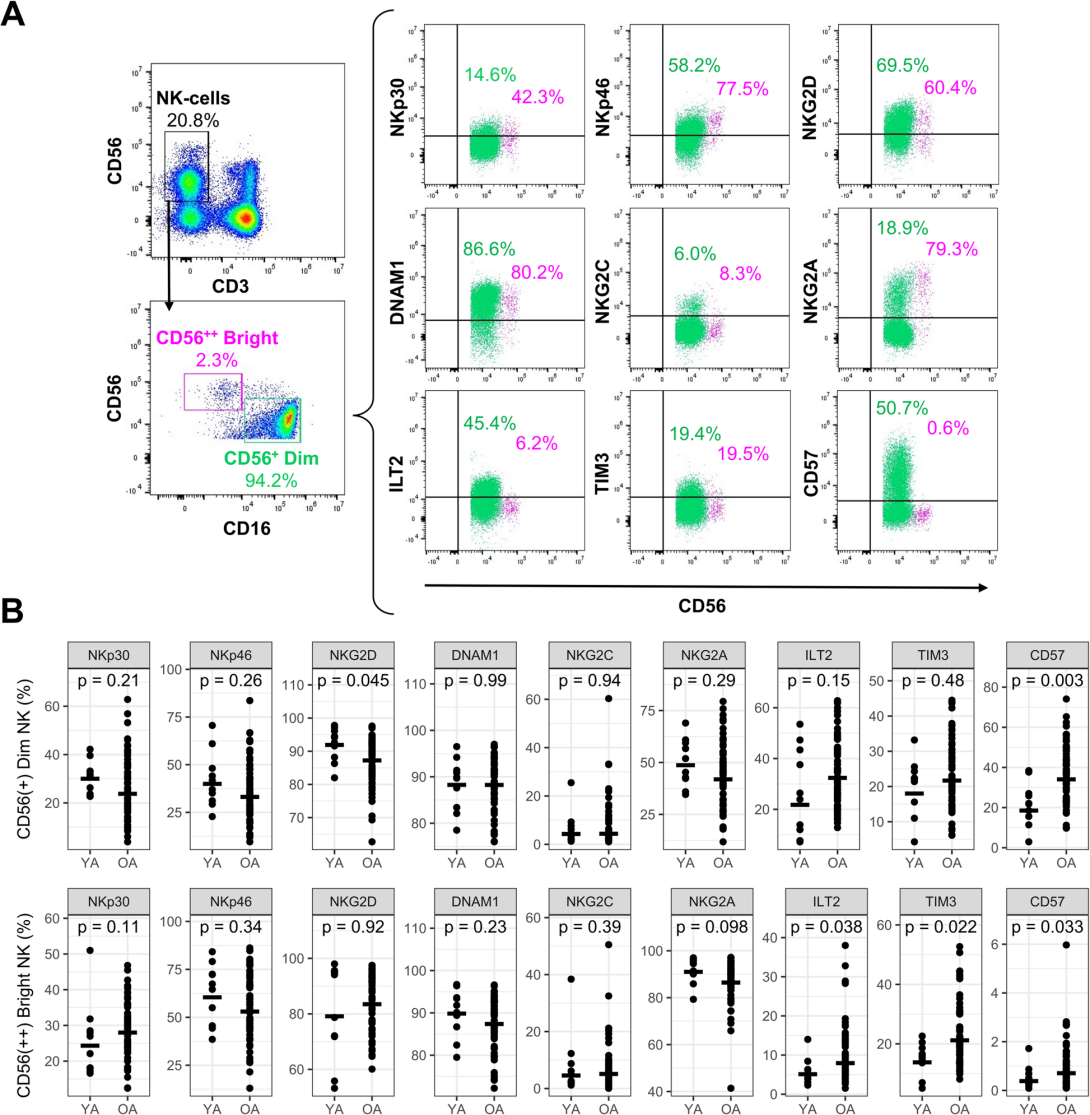
NK cell phenotype is altered with age. In the follow-up cohort (young [YA], *n* = 10; older [OA], *n* = 63), **A** NK-cells (CD56^+^CD3^−^ PBMCs) were identified by flow cytometry and gated into dim (CD56^+^; coloured green) and bright (CD56.^++^; coloured pink) subsets; the proportion of each subset expressing various phenotypic markers are shown for a single representative older adult. **B** Marker positivity in young and older adults, along with the geometric mean for each subset, and differences evaluated by Wilcoxon rank-sum test (ie. p)

Given recent findings suggesting that NK-cells support influenza vaccine responses [34, 35], we sought to determine a potential role for NK-cells in the relationship between inflammatory mediator levels and antibody responses. In a follow-up cohort of 63 older and 10 younger adults (Supplementary Table 3), pre-vaccination peripheral blood mononuclear cells (PBMCs) were gated into CD56^++^ Bright or CD56^+^ Dim NK-cell subsets, and the expression of the phenotypic markers NKp30, NKp46, NKG2D, DNAM1, NKG2C, NKG2A, ILT2, TIM3, and CD57 was measured (Fig. 3A). The frequency of NK-cells relative to total lymphocytes was significantly higher in older adults (14.6% ± 6.6 vs. 8.9% ± 3.4, *p* = 0.001), as was the ratio of the CD56^+^ Dim to CD56^++^ Bright subset (26.0 ± 16.3 vs. 12.2 ± 7.6, *p* = 0.011). Regarding the frequency of particular NK-cell phenotypic subsets, NKG2D^+^ and CD57^+^ Dim NK-cells were significantly lower and higher in older adults, respectively, while ILT2^+^, TIM3^+^ and CD57^+^ NK Bright cells were significantly higher in older adults (Fig. 3B).

**Fig. 4:**
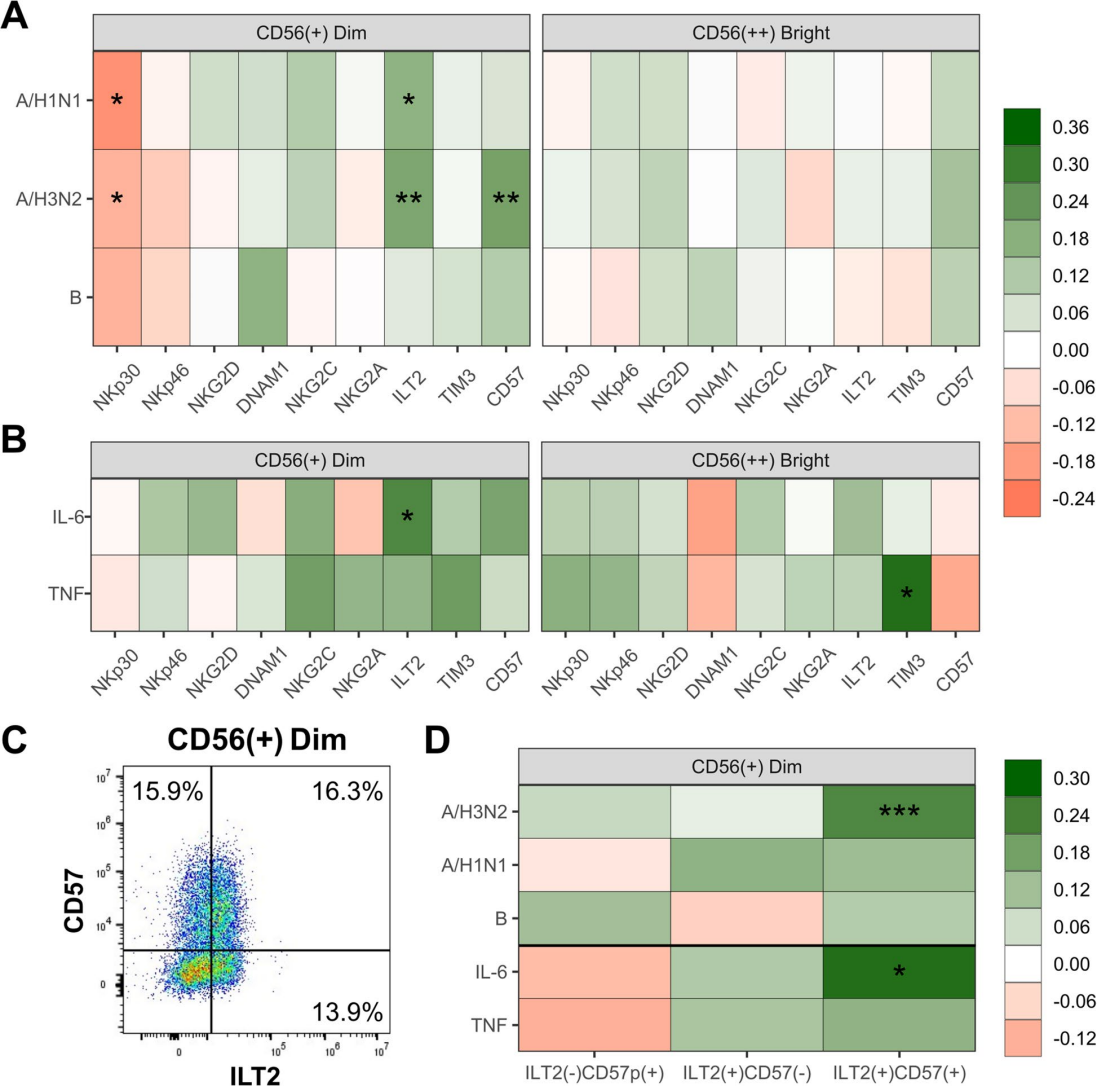
The frequency of ILT2^+^CD57^+^ CD56^+^ Dim NK-cells are significantly associated with both A/H3N2 vaccine antibody responses and pre-vaccination plasma IL-6 levels in older adults from the follow-up cohort (*n* = 63). **A** 4-week antibody responses were regressed on the frequency of positivity for different CD56^+^ Dim and CD56^++^ Bright NK-cell phenotypic markers and adjusted coefficients for each marker are shown. **B** Phenotype marker positivity was regressed on TNF or IL-6 and adjusted coefficients for each mediator are shown. **C** The geometric mean of the frequency of ILT2^−^CD57^+^, ILT2^+^CD57^+^ and ILT2^+^CD57.^−^ Dim NK-cells are shown, and overlayed on a representative participant dot plot. **D** Coefficients for each ILT2/CD57 subset relative to antibody titres (upper), and inflammatory mediators relative to subset frequency (lower) are shown. For **A**, **B** and **D**: antibody titres, inflammatory mediator levels and phenotype marker positivity were standardized and natural-log transformed to facilitate cross-comparison. Further, each phenotypic marker and inflammatory mediator were run in separate models, and the effect size and significance of coefficients for each fixed effect of interest (ie. marker or inflammatory mediator) are indicated by colour and asterisk (***, *p* < 0.001; **, *p* < 0.001; *, *p* < 0.05), respectively

To investigate whether NK-cell frequency was associated with vaccine antibody responses, 4-week antibody titres for young standard-dose recipients and older high-dose recipients were regressed against NK-cell subset frequencies, adjusting for baseline titre values, age, sex and recruitment site. Although no associations were observed in younger adults (data not shown), the phenotype of Dim NK-cells was significantly related to post-vaccination A/H1N1 and A/H3N2 antibody titres in older participants. Specifically, titres decreased 0.13- to 0.20-SD units for every 1-SD increase in NKp30 positivity, and increased 0.17- to 0.21-SDs with ILT2 or CD57 positivity (Fig. 4A). No significant associations with antibody responses in older adults were observed for overall NK-cell frequency or the ratio of Dim to Bright cells (data not shown). To evaluate whether NK-cell frequency was associated with systemic inflammation, the positivity of phenotypic markers on Dim and Bright NK-cells was regressed on plasma TNF or IL-6 levels in older adults. Here, two significant associations were observed: for every 1-SD increase in IL-6, the frequency of ILT2^+^ Dim NK-cells increased (standardized β [95% CI] = 0.27 [0.01, 0.54), while a similar increase in TNF was associated with increased TIM3^+^ Bright cells (0.33 [0.08, 0.58]) (Fig. 4B). Given that both the ILT2^+^ and CD57^+^ NK-cell subsets were higher in older adults (Fig. 3B), and were also associated with influenza A antibody titres post-vaccination (Fig. 4A), we sought to determine whether dual expression of ILT2 and CD57 on Dim NK-cells was more strongly associated with antibody titres or systemic inflammation. Indeed, double-positive ILT2^+^CD57^+^ Dim NK-cells, which make up roughly one-third of ILT2^+^ or CD57^+^ cells (Fig. 4C), were significantly associated with both post-vaccination A/H3N2 antibody titres (0.23 [0.10, 0.35]) and plasma IL-6 (0.28 [0.012, 0.54]), while the frequencies of ILT2^+^CD57^−^ or ILT2^−^CD57^+^ cells exhibited no significant associations (Fig. 4D).

### ILT2+CD57+ Dim NK-cells as a mediator in the relationship between IL-6 and A/H3N2 vaccination titres

**Fig. 5:**
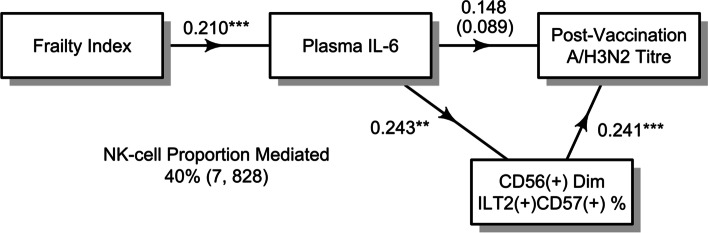
Causal mediation analysis to describe the relationship between frailty, IL-6 levels and the frequency of ILT2^+^CD57.^+^ Dim NK-cells with post-vaccination A/H3N2 antibody titres in older high-dose recipients. The association between the frailty index and IL-6 (β = 0.210) represents the unadjusted coefficient, estimated in older high-dose recipients from the 4-year trial (*n* = 289). Associations between IL-6 and NK-cell frequency (β = 0.243) and NK-cell frequency and post-vaccination antibody titres (β = 0.241) were estimated in the follow-up cohort (*n* = 63) and represent the unadjusted coefficient and the age, sex, site, baseline antibody titre and IL-6 adjusted coefficient, respectively. In the lower left, the proportion (95% confidence interval) that NK-cell frequency mediates the relationship between IL-6 and post-vaccination antibody titres is shown, and in the upper right, the total effect (ie. 0.148) and direct effect of IL-6 (ie. 0.089) is shown. All measures were standardized, while IL-6, NK-cell frequency and antibody titres were additionally natural-log transformed. Asterisks denote significance (***, *p* < 0.001; **, *p* < 0.01)

The observed associations for ILT2^+^CD57^+^ Dim NK-cells with both plasma IL-6 and A/H3N2 antibody titres suggests that this subset may be acting as a mediator along the pathway connecting systemic inflammation and post-vaccination antibody responses in older high-dose recipients. Given our previous work [10], this may actually be part of a larger biological mechanism that is driven by frailty. To test these hypotheses, we first performed causal mediation analysis, where the direct effect of plasma IL-6 on post-vaccination A/H3N2 titres (ie. IL-6 → A/H3N2 titres) and the indirect effect mediated through ILT2^+^CD57^+^ Dim NK-cell frequency (ie. IL-6 → ILT2^+^CD57^+^  → A/H3N2 titres) was calculated and compared. Here, the indirect effect was observed to be significant (0.059 [0.013, 0.13]), as was the proportion of the total effect mediated by ILT2^+^CD57^+^ Dim NK-cells (40% [7, 828]) (Fig. 5). Taken together with the significant positive correlation between frailty and plasma IL-6 observed in the parent vaccine trial (Figs. 1  Fig. 5), these results suggest that ILT2^+^CD57^+^ Dim NK-cells, increased by frailty-related inflammation, support the induction of antibodies against A/H3N2 following high-dose seasonal influenza vaccination in older adults. This is further supported by a similar mediation analysis in which the ILT2^+^CD57^+^ Dim NK-cell frequency significantly mediates (40% [16, 202]) the effect of frailty on post-vaccination A/H3N2 antibody titres (total effect = 0.16 [0.057, 0.35]; direct effect = 0.099 [-0.014, 0.29]) (Supplementary Fig. 4).

## Discussion

Using data and samples from our four-year randomized trial, we have shown that circulating levels of TNF, IL-6 and CRP are significantly associated with post-vaccination antibody titres in older adults receiving the high-dose influenza vaccine, and that titres are significantly higher in those with elevated levels of both TNF and IL-6, regardless of vaccine dose. In a smaller cohort of young adults, IL-6 was positively associated with A/H3N2 antibody titres, and inversely associated with PBMC IFN-γ secretion following live virus challenge. We have reported these associations in terms of Cohen’s d [36], all of which were small to medium effect sizes. Although it was somewhat unexpected to find that antibody responses increase with increasing systemic inflammation, especially considering previous work on the varicella-zoster virus [29] and standard-dose influenza vaccine [37], it aligns perfectly with our previous observation from this cohort where we showed that post-vaccination antibody titres increase with frailty, an inflammatory-related syndrome [32], in older, high-dose recipients [10]. The question remains, why systemic inflammation, normally a risk factor of adversity, would increase vaccine antibody responses, and why this association is much less apparent following standard-dose vaccine?

Administering high-dose instead of standard-dose vaccine has been shown to be an effective strategy for reducing serious outcomes of influenza in older adults [38]. This is largely believed to be related to the increased production of neutralizing antibodies, which we have shown to rival that of young adults [10]. These improvements are likely related to an improved elicitation of ICOS-expressing T follicular helper cells (Tfh) [39], which further drives the generation of vaccine-specific plasmablasts [40]. Although plasmablast generation and thus short-term antibody levels increase, long-lived B-cell frequency, T-cell cytokine responses, and overall persistence of vaccine-induced antibodies is apparently similar to what is seen in standard-dose vaccine recipients [40]; these findings of others align with our previous findings [10, 11]. Interestingly, IL-6 has also been shown to drive the generation of both Tfh cells and plasmablasts in animal models. Yousif and colleagues showed that IL-6-deficient mice exhibit poor induction of antibodies when challenged with hemagglutinin, which is mechanistically related to the differentiation of plasmablasts via IL-6 trans-signalling [41]. Harker and colleagues showed that the induction of IL-6 in late phases of chronic viral infection drive Tfh cell generation and germinal centre responses [42], which is also mediated through IL-6 trans-signalling [43]. Taken together, this body of evidence may explain why the combination of high-dose vaccine and elevated systemic inflammation is correlated with improved initial antibody responses in older adults, and only those standard-dose recipients with the highest TNF and IL-6 exhibit any association. The fact that young standard-dose recipients also exhibited a positive correlation between IL-6 levels and elevated antibody responses to A/H3N2 suggests that this “inflammatory-adjuvant” effect may wane with age, and that additional stimulation by high-dose vaccine may act to enhance this effect.

Besides being an important component of the immune response to influenza infection [44, 45], recent findings suggest a potential role of NK-cells in influenza vaccine responses. Indeed, NK-cells homing to draining lymph nodes following influenza vaccination were shown to support strong antibody responses [34], and other findings suggested that pre-vaccination CD57^+^ NK-cell frequency correlates with antibody responses [35]. Hence, to further understand the relationship between systemic inflammation and antibody responses, we performed a phenotypic screen of NK-cells in a follow-up cohort of young standard-dose and older high-dose vaccine recipients. As expected, we found an expansion of CD56-Dim NK-cells concomitant with a reduction of CD56-Bright NK-cells, resulting in an increase of total NK-cell frequency with age [46, 47]. In accordance with other studies [46, 48], the analysis of NK-cell activating and inhibitory receptors revealed an alteration of NK-cell phenotype with age, including (among others) an increased frequency of CD57^+^ NK cells in older adults. Importantly, we found that the frequency of ILT2^+^CD57^+^ CD56-Dim NK-cells was positively associated with both IL-6 levels and post-vaccination A-lineage titres, and through causal mediation analysis, that the frequency of this subset mediated a significant proportion of the association of IL-6 levels with A/H3N2 titres. The expression of CD57 is widely regarded as a marker of NK-cell maturation, which tends to become more frequent with age and coincides with an increase in ILT2 (also known as LIR-1 and LILRB1) and a decrease in NKp30 [49, 50], all of which we documented in the current study. Although this mature subset has been shown to be less proliferative and less responsive to cytokines, a higher proportion produce IFN-γ [51], which is a purported to be the mechanism by which NK-cells stimulate lymph node dendritic cells, and thereby antibody induction following influenza vaccination [34]. Alternatively, a greater proportion of mature NK-cells would also imply a reduced frequency of less mature (ie. CD57^−^) NK-cells, which have been suggested to contribute to the immunopathology of inflammatory disorders, such as psoriasis and rheumatoid arthritis [50]. The specific role of enhanced NK-cell ILT2 expression in vaccine responses is also not well described. ILT2 mainly acts as an inhibitory receptor on NK-, B- and T-cells, repressing cellular function following interaction with MHC-II [52]. Previous work has shown it to be increased on NK-cells following HPV vaccination, although it did not appear to impact IFNγ production by PBMCs upon restimulation [53]. While it is not clear whether NK-cell ILT2 plays a functional role in our context, given that NK-cells have also been implicated in the suppression of antibody responses via the suppression of both Tfh cells and plasmablasts [54], inhibition of NK-cell activity via ILT2 may in turn result in improved immunogenicity. Regarding the relationship between CD57^+^ NK-cells and IL-6, some studies have described increased frequencies of T-cells expressing CD57 along with elevated plasma levels of IL-6 [55, 56]. It is possible that additional inflammatory factors (or complex interactions with inflammatory factors), potentially related to frailty, are also involved. Clearly, further exploration into the role of CD57 and ILT2 expressing NK-cells following vaccination as well as how NK-cell phenotype relates to IL-6 and inflammation in general are warranted.

Our study featured a number of strengths. First, our associations with systemic inflammation are based on a large 4-year vaccine dose-randomized trial in well-characterized older adults, and a smaller cohort of younger adults, with clinical data collection including a robust frailty measure. Second, we assessed three different commonly-studied measures of inflammation as well as antibody responses against three viral (sub)types and cell-mediated responses for two effector cytokines. Lastly, we performed a broad characterization of NK-cell phenotype and used causal mediation analysis to investigate a possible role for NK-cells in the pathway from systemic inflammation to antibody titres. Given the nature of our study design we were limited in our ability to conclude true causation in the mechanistic pathway where frailty-related inflammation (ie. IL-6) leads to improved antibody responses via increased ILT2^+^CD57^+^ NK-cells, and likely enhanced lymph node Tfh cell and plasmablast development. Further, given the complexity of the frailty-inflammation relationship [32, 57–60], it is possible that another inflammatory factor, or even a group of factors, induced by frailty is promoting the NK-cell mediation relationship we have reported.

## Conclusions

In summary, we provide evidence that levels of systemic inflammatory factors improve initial antibody responses to seasonal influenza vaccines. In older adults, this association is limited to high-dose vaccine recipients and standard-dose recipients featuring the highest levels of inflammation, and may be mediated through the activity of ILT2^+^CD57^+^ Dim NK-cells. Frailty appears to be an important upstream factor in this pathway, although further study of the pathophysiological consequences of frailty, especially in the context of immunity, is needed in order to make definitive conclusions. Further, while our results suggest that “inflammaging” may increase vaccine immunogenicity in older adults, it is yet to be determined whether this enhancement contributes to improved protection against influenza disease.

## Methods

### Study design

The current study was a secondary analysis of data and biosamples from a double-blind randomized controlled trial to compare the immunogenicity of a trivalent high-dose versus quadrivalent standard-dose formulation of the split-virus influenza vaccine (Fluzone, Sanofi Pasteur) in community-dwelling older adults (ClinicalTrials.gov: NCT02297542); the design and protocol have been previously published [10]. A cohort of young, healthy adults receiving standard-dose vaccination were also recruited and followed the same protocol. Over four consecutive influenza seasons (October 2014 – April 2015, October 2015 – April 2016, October 2016 – April 2017, and October 2017 – April 2018), 612 older adults and 79 young adults were enrolled, vaccinated and provided blood for immune testing. However, since participants were allowed to re-enroll in subsequent seasons, the number of unique individuals over the four years was 246 and 40, for older and younger subjects, respectively. In each year, older participants were randomized 1:1 to receive either standard or high-dose vaccine (Fluzone, Sanofi Pasteur) at each study site, and blood samples were collected at the pre-vaccination and 4-, 10- and 20-week post-vaccination visits. In addition to the parent four-year trial, a follow-up cohort of 63 older and 10 young adults were recruited for the subsequent influenza season (October 2018 – April 2019) and were the focus of the flow cytometry based studies. These participants followed the same protocol, only all older adults received high-dose vaccine, instead of being randomized. For the current study, only pre-vaccination and 4-week post-vaccination samples and related data were considered. The study protocol was approved by the Institutional Review Board of the University of Connecticut Health Centre (UCHC) and the Health Sciences North Research Ethics Board and all study participants provided written informed consent to participate in the study.

### Sites and study participants

Adults aged 65 years and older were recruited at the Health Sciences North Research Institute (HSNRI) from the community of Greater Sudbury, Ontario, Canada, and at UConn Health through the University of Connecticut Center on Aging Recruitment Core from the communities belonging to and surrounding Hartford, Connecticut, USA. Inclusion criteria included: willing to receive the influenza vaccine in the current season, at least 65 years old and were vaccinated in the previous influenza season. Exclusion criteria included: known immunosuppressive disorders or medications including prednisone in doses > 10 mg/day, or a previous severe reaction to the vaccine due to egg, latex, or thimerosol allergies. Research coordinators ensured that vaccinations were scheduled at least 2 weeks after any acute respiratory illness.

Following informed consent, study participants were characterized according to demographics, chronic medical conditions, and functional impairments. BMI was calculated using weight and height measurements derived from a physician’s scale and analyzed in relation to a clinically meaningful difference of 2 kg/m^2^ [61]. A frailty index (FI) was calculated based on 40 items representing accumulated health deficits across multiple systems, which has been previously validated in influenza studies [62–64], and has been previously employed in studies of this trial [10, 11]. The FI counts the proportion of health deficits an individual has relative to the total number considered (ie. 40), and generally ranges between 0 and 0.7 [65, 66]; a difference of 0.03 in the FI has been shown to be clinically meaningful [67].

### Antibody and cell-mediated immune response measures

Hemagglutination-inhibition (HAI) antibody titres were quantified using previously-described standard methods [10, 68]. Influenza types used for HAI testing were as follows: Year 1, A/Texas/50/2012, A/California/7/2009 and B/Massachusetts/2/2012; Year 2, A/Switzerland/9715292–2013, A/California/7/2009 and B/Phuket/3073/2013; Year 3, A/Hong Kong/4801–2014, A/California/7/2009 NYMC X-179A and B/Brisbane/60/2008; and Year 4, A/HongKong/4801/2014, A/Michigan/45/2015 and B/Brisbane/60/2008. For the follow-up cohort (ie. Year 5), A/HongKong/4801/2014, A/Michigan/45/2015 and B/Colorado/06/2017 were used. Laboratory testing was conducted after each study year, and participant serum was randomized before plating.

IFN-γ and IL-10 concentrations were assessed using previously described standard operating procedures [11, 69]. Briefly, thawed PBMCs were stimulated with sucrose-gradient purified, live influenza virus (A/Victoria/3/75; Charles River, MA, USA) at a multiplicity of infection (MOI) of 2 in AIM V media (Life Technologies, ON, Canada) and incubated at 37 °C/5% CO_2_ for 20 h; this strain was chosen in order to increase consistency from year to year and because it contains both hemagglutinin and internal protein (matrix 1 and nucleoprotein) peptide sequences that are shared and thus cross-reactive across all A/H3N2 strains. Supernatants and lysates were collected and stored at -80 °C until assay measurement at the end of each study year. Concentrations of IFNγ and IL-10 were measured in supernatants by multiplexed bead ELISA (Millipore, ON, Canada) and reported as pg/ml.

### Inflammatory mediators and other serological measures

TNF and IL-6 were measured in participant plasma pre-vaccination using the Ella Automated Immunoassay System and Simple Plex 2^nd^ generation Human TNF and IL-6 cartridges (R&D Systems, MN, USA). Samples were randomized prior to plating, and a single biological replicate was included on each cartridge to assess inter-assay variability. The inter-assay CV was 4% for TNF, and 14% for IL-6 and both measures are presented as pg/ml. CRP was measured by immunoturbidimetric method at the Health Sciences North clinical laboratory; given the lower limit of detection for this assay (5 mg/L, as opposed to high-sensitivity CRP), participants were categorized as low or undetectable (< 5 mg/L), medium (5–10 mg/L) and high (10 or more mg/L), the latter being the upper limit of normal [70]. CMV serostatus was determined in serum using a CMV IgG ELISA kit (Genesis Diagnostics Inc., Cambridgeshire, UK) according to the manufacturer’s instructions.

### Flow cytometry analyses

Following thawing, PBMCs from the baseline collection (0.5 × 10^6^ cells) were resuspended in flow cytometry staining buffer (PBS, 2% FBS, 2 mM EDTA, 0.01% NaN3) and incubated first with Fixable Viability Dye (FvD) eFluorTM 780 (eBioscience, CA, USA) and 10% heat-inactivated human AB serum (Sigma-Aldrich, MO, USA), for 15 min at 4 °C. After washing with flow cytometry staining buffer, PBMCs were then stained with CD3-AF700, CD16-FITC, CD56-PercpCy5.5, NKp30-BV785, NKp46-BV510, NKG2A-PeCy7, NKG2D-PE/Dazzle, DNAM-1-APC, ILT2-PE, TIM-3-BV421, CD57-BV605 (Biolegend, CA, USA) and NKG2C-BV650 (BD Biosciences, NJ, USA), for 20 min at 4 °C. Samples were acquired with a CytoFLEX flow cytometer (Beckman Coulter, CA, USA) running CytExpert software v.2.1 (Beckman Coulter, CA, USA) immediately after staining.

Analysis of flow data was performed using FlowJo v.10.1.5. Initially, the duplicates were removed via progressive gating on FSC-area vs. FSC-height and SSC-area vs. SSC-height. Next, dead cells were excluded by considering only FvD-negative cells and then, a morphological gate (FSC-area vs. SSC-area) was used to identify the lymphocyte population. Total NK cells were defined as CD3^−^CD56^+^ cells and further subdivided into CD56^+^ Dim CD16^+^ and CD56^++^ Bright CD16^−/low^ NK cell subsets. Each marker was individually analyzed on NK cell subsets (total, CD56-Dim and CD56-Bright) by plotting against CD56 and then markers were collectively analyzed by plotting each other.

### Statistical Analysis

#### Summary statistics and bivariate analysis

Continuous demographic variables were summarized as the mean and standard deviation, while inflammatory mediators were summarized using the mean, standard deviation, median and range. Vaccine response and NK-cell frequency measures were described by geometric mean and 95% confidence interval. All categorical data were summarized as the count and frequency. Bivariate comparisons between age groups were performed by either Wilcoxon rank-sum or chi-square test.

#### Primary regression analyses with vaccine outcomes

To investigate the association between inflammatory mediator levels and participants’ demographics, mixed model regression was employed using the R package “lme4” with the Nelder-Mead optimizer. For TNF and IL-6, standardized (mean = 0, standard deviation = 1) natural-log transformed values were regressed on fixed effects for age, sex, vaccine dose, BMI, CMV serostatus and FI, and random intercepts for site, year and participant; dose and FI were not included in models for young adults. For CRP, parallel logistic regression models were employed, one for the comparison of the medium and low concentration groups (ie. high concentration participants were removed), and the other for the high and low concentration groups (ie. medium concentration participants were removed); fixed and random covariates were included as above. To investigate the association between inflammatory mediator levels and vaccine response measures, standardized natural-log transformed 4-week values for antibody titres or IFN-γ or IL-10 secretion were regressed on fixed effects for the natural-log transformed pre-vaccination value and the standardized natural-log transformed pre-vaccination value for TNF or IL-6, or CRP, and random intercepts for site, year and participant; hence only 1 inflammatory mediator was included per model. Multivariable models in which all three mediators were fit as fixed effects were also performed in order to assess independent effects; the variance inflation factor for all three mediators were 1.25 or less. Joint effects were estimated using interaction analysis on an addictive scale [71], where levels of TNF and IL-6 were categorized as high (≥ 0.5-SDs from sample mean), medium (-0.5 to 0.5-SDs from the mean), or low (≤ -0.5-SDs from the mean), and four categories were derived: TNF-low/medium + IL-6-low/medium, TNF-low/medium + IL-6-high, TNF-high + IL-6-low/medium, and TNF-high + IL-6-high. This categorical variable was modelled as a fixed effect in place of individual mediators, as described above. Models for older adults included the fixed effects of age, sex, vaccine dose and a dose by inflammatory mediator interaction, whereas for young adults, models also included the fixed effects of sex and BMI; these additional factors were found to improve model fit according to the Akaike information criterion (AIC). For all regression models employed in the current study, results are presented as the coefficient (or odds ratio) and 95% confidence interval and participants with missing data were removed from the analysis. Coefficients for standardized, continuous outcomes are to be interpreted in terms of 1-SD unit changes, or in other words, Cohen’s d [36].

#### Secondary analyses related to vaccine outcomes

To elucidate whether the association of inflammatory mediators and antibody responses were independent of frailty in high-dose older adult recipients, coefficients from the aforementioned models were compared to models in which the fixed effect of frailty was included. We also investigated whether sex modified the association between vaccine responses and inflammatory mediators. Here, we ran the aforementioned models (only for TNF and IL-6) in male and female strata and presented coefficients for young and older adults, by dose. To test the modifying effect of sex, we performed models that included a sex by TNF or IL-6 interaction term in age- and dose-specific strata.

#### Analysis of blood NK cells in the follow-up cohort and mediation analyses

Associations between NK-cell frequency and antibody responses were evaluated using linear regression using a similar approach as described above, only all models were adjusted for the fixed effects of age, sex and site, and no random effects were included. This was also the case for associations between plasma TNF or IL-6 with NK-cell frequency (dependent variable). In both cases, NK-cell measures were standardized and natural log-transformed.

Causal mediation analysis was used to determine whether NK-cell frequency mediated the effect of plasma IL-6 on A/H3N2 antibody responses in older adults from the follow-up cohort; this approach was similarly used for frailty as the exposure variable. Total, direct and indirect effects and the proportion mediated were estimated using the R package ‘mediation’ [72], with bias-corrected and accelerated (BCa) 95% confidence intervals calculated after 5000 simulations.

All analyses were performed in the R environment (v3.6).

## Supplementary Information

**Additional file 1: Table S1.** Summary of antibody and cell-mediated immune responses pre- and 4-weeks post-influenza vaccination. **Table S2.** Results from multivariable analysis of vaccine antibody responses in older adults where plasma TNF, IL-6 and CRP were simultaneously included as fixed effects. **Table S3.** Summary of participants enrolled in the follow-up cohort, including pre-vaccination levels of inflammatory mediators and antibody responses pre- and 4-weeks post-influenza vaccination. **Figure S1.** Associations between inflammatory mediator levels and antibody responses of high-dose vaccine recipients are independent of frailty. **Figure S2.** Joint effects of TNF and IL-6 in the association with post-vaccination antibody titres in standard- dose (SD) and high-dose (HD) older recipients. **Figure S3.** Sex-stratified models of systemic inflammation and the response to influenza vaccination in young (YA) and older (OA) adults vaccinated with either standard (SD) or high (HD) dose vaccine. **Figure S4.** ILT2^+^CD57^+^ Dim NK-cells mediate a significant proportion of the effect of frailty on post-vaccination A/H3N2 antibody titres in older high-dose recipients.

## Data Availability

The data sets generated and analyzed during the current study are available from the corresponding author on reasonable request.

## Abbreviations

AIC: Akaike information criterion
BMI: Body mass index
CMV: Cytomegalovirus
CRP: C-reactive protein
FI: Frailty index
FvD: Fixable viability dye
HAI: Hemagglutination-inhibition
IFN-γ: Interferon-γ
IL-6: Interleukin-6
IL-10: Interleukin-10
MOI: Multiplicity of infection
NK: Natural killer
PBMC: Peripheral blood mononuclear cell
TNF: Tumor necrosis factor
