# Supplementary material for: Markers of systemic inflammation are positively associated with influenza vaccine antibody responses with a possible role for ILT2(+)CD57(+) NK-cells
Source: Immun Ageing. 2022 May 26;19:26. doi: 10.1186/s12979-022-00284-x (PMC9134679; doi:10.1186/s12979-022-00284-x)
Supplement: Supplementary file 1 — Additional file 1: Table S1. Summary of antibody and cell-mediated immune responses pre- and 4-weeks post-influenza vaccination. Table S2. Results from multivariable analysis of vaccine antibody responses in older adults where plasma TNF, IL-6 and CRP were simultaneously included as fixed effects. Table S3. Summary of participants enrolled in the follow-up cohort, including pre-vaccination levels of inflammatory mediators and antibody responses pre- and 4-weeks post-influenza vaccination. Figure S1. Associations between inflammatory mediator levels and antibody responses of high-dose vaccine recipients are independent of frailty. Figure S2. Joint effects of TNF and IL-6 in the association with post-vaccination antibody titres in standard- dose (SD) and high-dose (HD) older recipients. Figure S3. Sex-stratified models of systemic inflammation and the response to influenza vaccination in young (YA) and older (OA) adults vaccinated with either standard (SD) or high (HD) dose vaccine. Figure S4. ILT2+CD57+ Dim NK-cells mediate a significant proportion of the effect of frailty on post-vaccination A/H3N2 antibody titres in older high-dose recipients. [file 12979_2022_284_MOESM1_ESM.docx]

# Supplementary Tables

**Supplementary Table 1:** Summary of antibody and cell-mediated immune responses pre- and 4-weeks post-influenza vaccination.

|  |  | **Older adults (SD)** | **Older adults (HD)** | **Young adults (SD)** |
| --- | --- | --- | --- | --- |
|  |  | **(N=316)** | **(N=296)** | **(N=79)** |
| **A/H1N1 (titre)** | **Pre-vax** | 43 (39.3, 46.9) | 41 (36.5, 45.1) | 102 (80, 129) |
|  | **4-week** | 68 (61.9, 74.5) | 111 (99.6, 124) | 162 (131, 202) |
| **A/H3N2 (titre)** | **Pre-vax** | 44 (39.6, 50.2) | 51 (44.7, 57) | 67 (52.5, 85.5) |
|  | **4-week** | 120 (106, 136) | 195 (171, 224) | 186 (150, 233) |
| **B (titre)** | **Pre-vax** | 40 (36.2, 43.2) | 36 (32.6, 39.7) | 64 (50, 84) |
|  | **4-week** | 66 (60, 73.4) | 91 (82.2, 101) | 130 (105, 160) |
| **IFN-γ (pg/ml)** | **Pre-vax** | 1195 (1074, 1329) | 1086 (968, 1224) | 1828 (1461, 2281) |
|  | **4-week** | 1272 (1147, 1418) | 1240 (1102, 1393) | 2142 (1713, 2695) |
| **IL-10 (pg/ml)** | **Pre-vax** | 12 (11, 12.9) | 12 (10.8, 12.7) | 19 (16.5, 21.9) |
|  | **4-week** | 19 (17.3, 20.1) | 25 (23, 27.5) | 28 (24.7, 31.9) |

Data summarized as the geometric mean and 95% confidence interval. SD, standard-dose; HD, high-dose.

**Supplementary Table 2:** Results from multivariable analysis of vaccine antibody responses in older adults where plasma TNF, IL-6 and CRP were simultaneously included as fixed effects.

|  | **scale(log(H1N1_V2))** | | | **scale(log(H3N2_V2))** | | | **scale(log(B_V2))** | | |
| --- | --- | --- | --- | --- | --- | --- | --- | --- | --- |
| *Predictors* | *Estimates* | *CI* | *p* | *Estimates* | *CI* | *p* | *Estimates* | *CI* | *p* |
| (Intercept) | -2.685 | -3.530, -1.840 | **<0.001** | -1.86 | -2.728, -0.991 | **<0.001** | -1.842 | -2.624, -1.059 | **<0.001** |
| V1 HAI [log] | 0.753 | 0.686, 0.821 | **<0.001** | 0.599 | 0.533, 0.664 | **<0.001** | 0.77 | 0.696, 0.844 | **<0.001** |
| Age | 0.001 | -0.009, 0.011 | 0.888 | -0.004 | -0.014, 0.006 | 0.405 | -0.009 | -0.019, 0.000 | 0.056 |
| Sex [Male] | -0.045 | -0.182, 0.093 | 0.525 | -0.071 | -0.219, 0.077 | 0.345 | -0.152 | -0.294, -0.009 | **0.037** |
| Dose [SD] | -0.455 | -0.573, -0.336 | **<0.001** | -0.338 | -0.479, -0.198 | **<0.001** | -0.397 | -0.527, -0.267 | **<0.001** |
| sl.TNF | 0.179 | 0.098, 0.261 | **<0.001** | 0.124 | 0.031, 0.217 | **0.009** | 0.189 | 0.102, 0.275 | **<0.001** |
| Dose [SD] * sl.TNF | -0.173 | -0.280, -0.066 | **0.001** | -0.123 | -0.252, 0.005 | 0.06 | -0.169 | -0.286, -0.051 | **0.005** |
| sl.IL6 | 0.022 | -0.058, 0.103 | 0.583 | 0.086 | -0.005, 0.176 | 0.064 | 0.115 | 0.030, 0.201 | **0.008** |
| Dose [SD] * sl.IL6 | 0.026 | -0.084, 0.136 | 0.638 | -0.046 | -0.178, 0.085 | 0.491 | -0.057 | -0.178, 0.063 | 0.352 |
| CRP [5-10] | 0.207 | 0.008, 0.407 | **0.042** | 0.019 | -0.215, 0.253 | 0.873 | 0.023 | -0.194, 0.240 | 0.833 |
| CRP [10+] | 0.302 | 0.019, 0.586 | **0.037** | 0.258 | -0.077, 0.592 | 0.131 | 0.176 | -0.134, 0.485 | 0.266 |
| Dose [SD] * CRP [5-10] | -0.219 | -0.486, 0.048 | 0.108 | 0.124 | -0.194, 0.443 | 0.444 | 0.01 | -0.283, 0.304 | 0.945 |
| Dose [SD] * CRP [10+] | -0.252 | -0.672, 0.168 | 0.239 | -0.216 | -0.713, 0.280 | 0.393 | -0.265 | -0.724, 0.194 | 0.258 |
| **Random Effects** | | | |  | | |  | | |
| σ^2^ | 0.29 | | | 0.45 | | | 0.37 | | |
| τ_00_ | 0.12 _ComboID_ | | | 0.09 _ComboID_ | | | 0.10 _ComboID_ | | |
|  | 0.00 _Year_ | | | 0.11 _Year_ | | | 0.02 _Year_ | | |
|  | 0.02 _Site_ | | | 0.00 _Site_ | | | 0.00 _Site_ | | |
| N | 231 _ComboID_ | | | 231 _ComboID_ | | | 231 _ComboID_ | | |
|  | 2 _Site_ | | | 2 _Site_ | | | 2 _Site_ | | |
|  | 4 _Year_ | | | 4 _Year_ | | | 4 _Year_ | | |
| Observations | 567 | | | 567 | | | 567 | | |
| Marginal R^2^ | 0.555 | | | 0.509 | | | 0.568 | | |

Post-vaccination antibody titres (ie. V2) and TNF/IL-6 were natural-log transformed and standardized (ie. “sl”), while sub-type specific pre-vaccination antibody titres (ie. V1 HAI) were only log-transformed. The reference category is denoted in square brackets, hence, coefficients for inflammatory mediators represent the estimate for high-dose participants. Significant p-values (ie. <0.05) are bolded.

**Supplementary Table 3:** Summary of participants enrolled in the follow-up cohort, including pre-vaccination levels of inflammatory mediators and antibody responses pre- and 4-weeks post-influenza vaccination.

|  | **Older adults [High-dose]** | **Young adults [Standard-dose]** |
| --- | --- | --- |
|  | **(N=63)** | **(N=10)** |
| **Age** | 78 (7.04) | 32 (4.86) |
| **Sex** |  |  |
| Female | 39.0 (61.9%) | 6.00 (60.0%) |
| Male | 24.0 (38.1%) | 4.00 (40.0%) |
| **BMI (kg/m²)** | 28 (5.24) | 30 (8.57) |
| **CMV serostatus** |  |  |
| Negative | 23.0 (36.5%) | 5.00 (50.0%) |
| Positive | 40.0 (63.5%) | 5.00 (50.0%) |
| **Frailty Index** | 0.088 (0.0637) |  |
| **Site** |  |  |
| HSNRI | 35.0 (55.6%) | 5.00 (50.0%) |
| UCHC | 28.0 (44.4%) | 5.00 (50.0%) |
| ***Inflammatory mediators*** | |  |
| **TNF (pg/ml)** |  |  |
| Mean (SD) | 11.3 (3.36) | 8.02 (1.85) |
| Median [Min, Max] | 11.0 [6.05, 22.9] | 7.68 [6.13, 11.9] |
| Missing | 1 (2%) | 0 (0%) |
| **IL-6 (pg/ml)** |  |  |
| Mean (SD) | 3.88 (4.86) | 1.88 (1.75) |
| Median [Min, Max] | 2.70 [0.598, 28.0] | 1.28 [0.512, 6.19] |
| Missing | 1 (2%) | 0 (0%) |
| ***Antibody titres*** |  |  |
| **A/H1N1 (pre-vax)** | 39 (31.7, 51) | 63 (26.4, 139) |
| **A/H1N1 (4-week)** | 73 (56.3, 95.4) | 92 (49.2, 160) |
| **A/H3N2 (pre-vax)** | 43 (31.9, 57.8) | 130 (62.8, 269) |
| **A/H3N2 (4-week)** | 91 (69, 120) | 197 (98.5, 368) |
| **B (pre-vax)** | 42 (33.5, 51.5) | 37 (25.5, 58.6) |
| **B (4-week)** | 89 (70.5, 111) | 72 (42.9, 121) |

Continuous demographic is summarized as the mean and standard deviation, while categorical data is the count and frequency; note, the frailty index was not measured in young adults. For antibody titres, data is summarized as the geometric mean and 95% confidence interval.

# Supplementary Figures


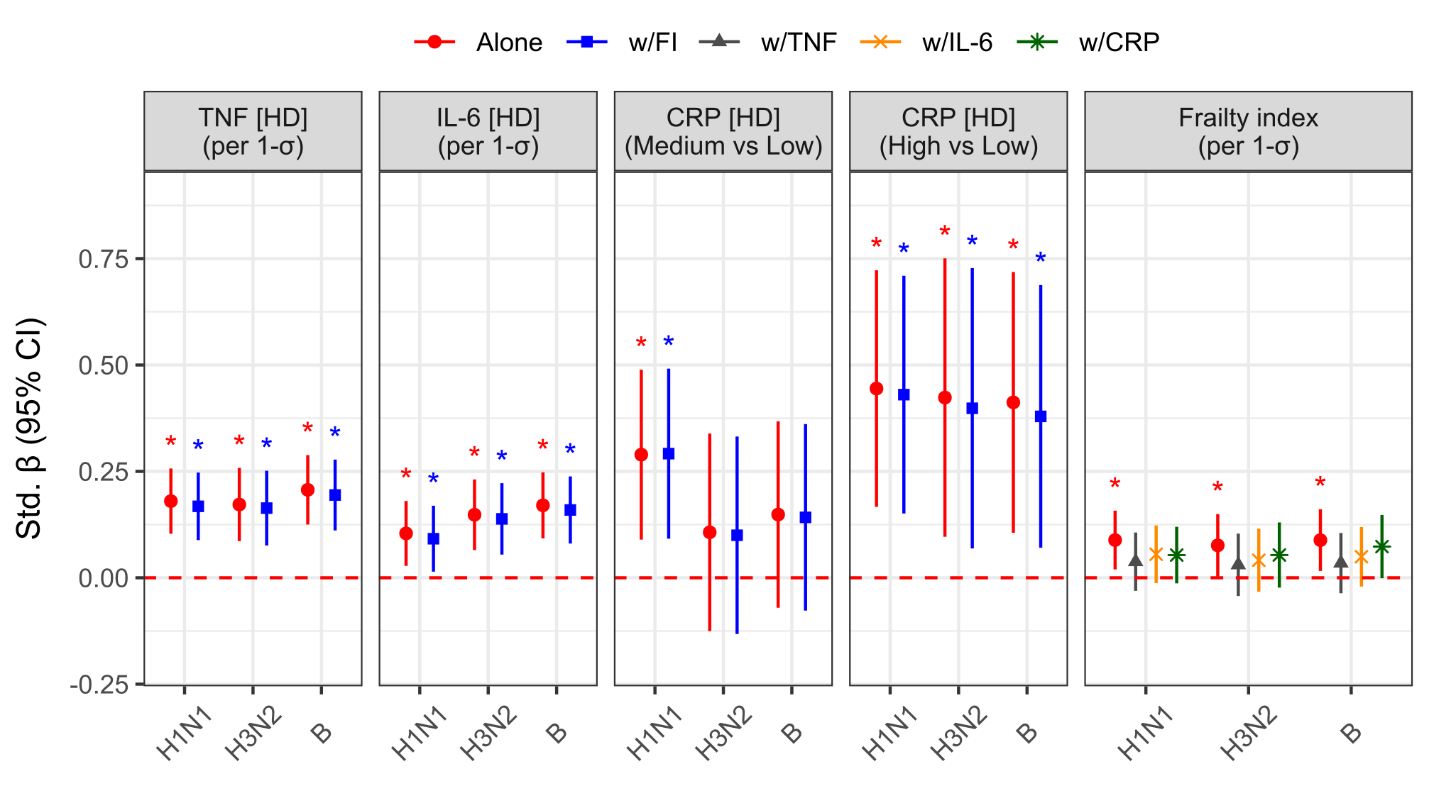


**Supplementary Figure 1:** Associations between inflammatory mediator levels and antibody responses of high-dose vaccine recipients are independent of frailty. In a series of base models, natural-log transformed antibody titres 4-weeks post-vaccination was regressed on natural log-transformed TNF or IL-6, CRP, or the frailty index (FI), adjusting for pre-vaccination antibody titres, age, sex, dose and dose x mediator and random intercepts for site, year and participant. FI was then added to each base model featuring an inflammatory mediator, and the resulting coefficients compared to that of the corresponding base model. Standardized coefficients (β) and 95% confidence intervals are presented for: TNF, IL-6, CRP and FI in base models (Alone); TNF, IL-6 and CRP when modelled with FI (w/ FI); and FI when modelled with TNF (w/ TNF), IL-6 (w/ IL-6), and CRP (w/ CRP). Coefficients are relative to the change or contrast described in brackets, and significance is indicated by an asterisk and when the 95% confidence interval does not cross the red, dotted line.


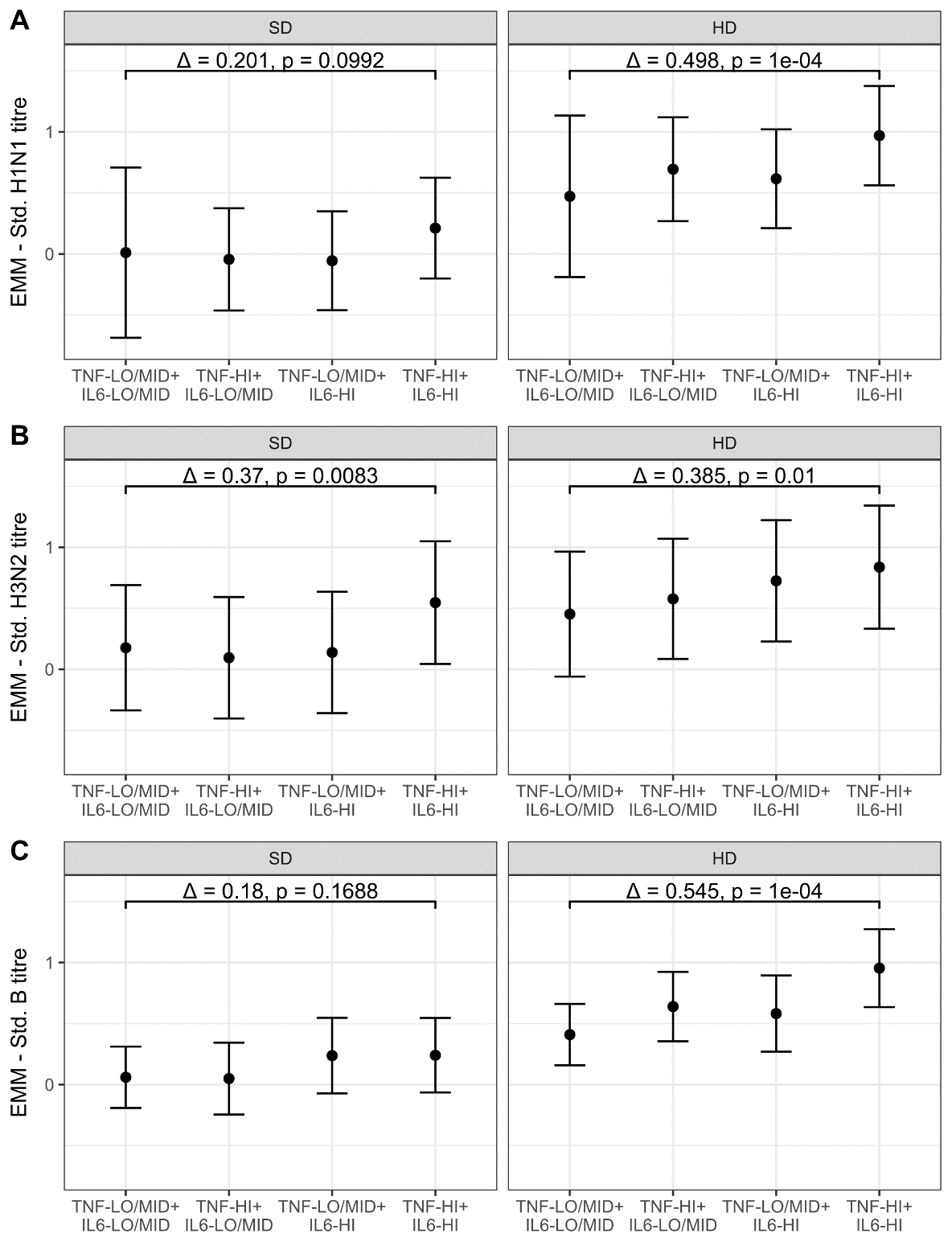


**Supplementary Figure 2:** Joint effects of TNF and IL-6 in the association with post-vaccination antibody titres in standard- dose (SD) and high-dose (HD) older recipients. Participant TNF and IL-6 levels were recoded as low/medium (LO/MID) or high (HI), and estimated marginal means (EMM) of post-vaccination A) A/H1N1, B) A/H3N2 and C) B antibody titres for four joint levels (ie. x-axis) were estimated using mixed model regression. EMMs are shown as the estimate and 95% confidence interval, and the contrast between TNF-HI+IL6-HI vs. TNF-LO/MID+IL6-LO/MID categories is shown as the difference in EMM (Δ) and p-value.
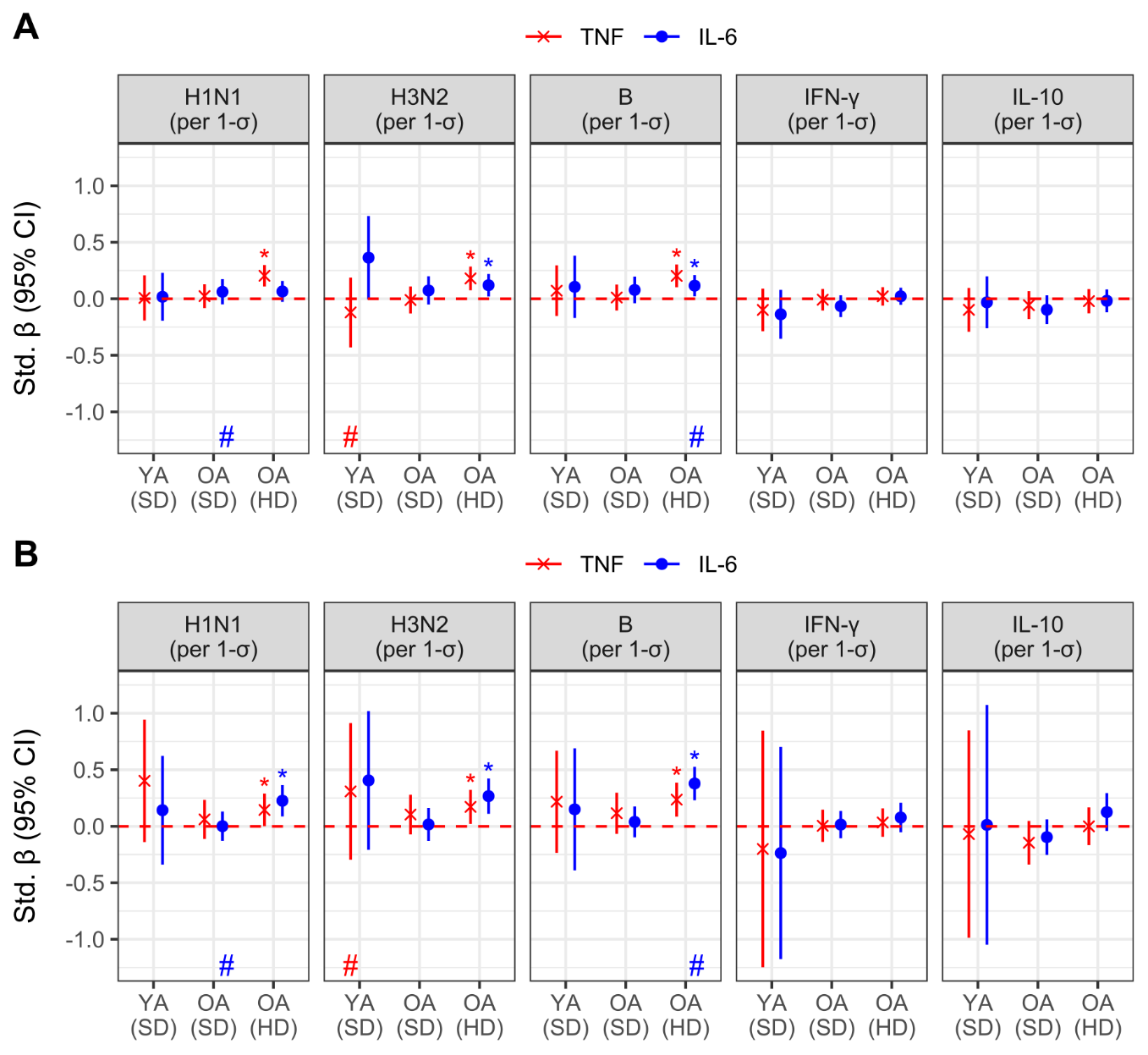


**Supplementary Figure 3:** Sex-stratified models of systemic inflammation and the response to influenza vaccination in young (YA) and older (OA) adults vaccinated with either standard (SD) or high (HD) dose vaccine. In either females (A) or males (B) natural log-transformed antibody titres against the seasonal circulating strain of influenza A/H1N1, A/H3N2 and B, and PBMC IFN-γ and IL-10 secretion in response to *ex vivo* A/H3N2 infection were measured at 4-weeks post-vaccination and regressed against pre-vaccination natural-log transformed TNF or IL-6, adjusting for pre-vaccination antibody/CMI response and additional covariates; both the outcome and TNF/IL-6 were standardized to facilitate cross-comparison. Standardized coefficients (β) and 95% confidence intervals are presented, and significance of TNF or IL-6 effect is indicated by an asterisk and when the 95% confidence interval does not cross the red, dotted line. Models featuring interactions between sex and TNF or IL-6 were also performed in dose-stratified analyses for YA and OA; significance of the sex interaction effect is denoted by a hash symbol (#) in panel A and B.


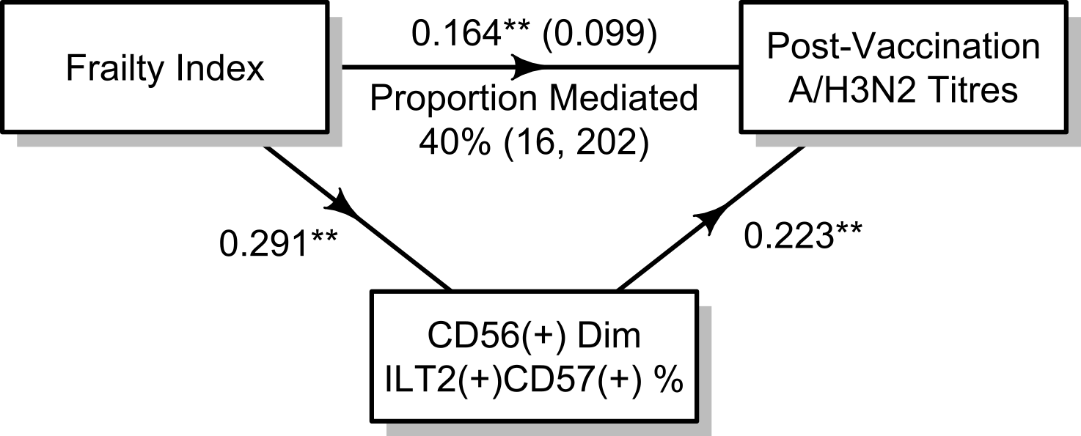


**Supplementary Figure 4:** ILT2^+^CD57^+^ Dim NK-cells mediate a significant proportion of the effect of frailty on post-vaccination A/H3N2 antibody titres in older high-dose recipients. Associations between frailty and NK-cell frequency (β=0.291) and NK-cell frequency and post-vaccination antibody titres (β=0.223) were estimated in the validation cohort (n=63) and represent the unadjusted coefficient and the age, sex, site, baseline antibody titre and frailty adjusted coefficient, respectively. The proportion (95% confidence interval) that NK-cell frequency mediates the relationship between frailty and A/H3N2 titres is shown, as is the total effect (ie. 0.164) and direct effect of frailty (ie. 0.099). All measures were standardized, while NK-cell frequency and antibody titres were additionally natural-log transformed. Asterisks denote significance (**, p<0.01).
